# Misdiagnosis of a Paediatric Fibrous Dysplasia of the Mandible

**DOI:** 10.7759/cureus.82872

**Published:** 2025-04-23

**Authors:** Retaj Alawadhi, Sood Alsairefi, Mishal M AlMutairi

**Affiliations:** 1 Medicine and Surgery, Royal College of Surgeons in Ireland, Dublin, IRL; 2 General Surgery, Royal College of Surgeons in Ireland, Dublin, IRL; 3 Otorhinolaryngology – Head and Neck Surgery, Farwaniya Hospital, Farwaniya, KWT

**Keywords:** chronic parotitis, craniofacial fibrous dysplasia, facial swelling, fibrous bone dysplasia, fibrous dysplasia (fd), monostotic fibrous dysplasia

## Abstract

Fibrous dysplasia (FD) is a chronic benign bone lesion, mainly affecting young individuals, that could be monostotic or polyostotic in nature leading to the replacement of normal bone with the excess proliferation of fibrous tissue. Patients are usually asymptomatic and are incidentally diagnosed after an oral imaging. The first symptom is typically the painless growth of the affected bone, presenting as facial asymmetry. Here, we present a case of an 18-year-old female patient with a recent diagnosis of right mandibular fibrous dysplasia who was misdiagnosed for the past seven years. The physical examination indicated the presence of hard right submandibular swelling, and imaging confirmed fibrous dysplasia of the right mandible with involvement of the right alveolar foramen and oedematous changes of the right masseter muscle. This case highlights the familiarity of fibrous dysplasia of the mandible in young patients and emphasizes the importance of careful investigation measures and multidisciplinary collaboration in achieving correct diagnosis and management for such challenging cases.

## Introduction

Fibrous dysplasia (FD) is a benign skeletal disorder caused by a post-zygotic missense mutation in the GNAS gene (Guanine Nucleotide-Binding Protein, Alpha-Stimulating), which encodes a protein that regulates cyclic AMP signaling, controlling bone formation and remodeling [1]. With a prevalence of 1 in 10,000 to 1 in 20,000, FD constitutes approximately 2.5-5% of benign bone lesions and 7% of benign bone tumors, with the monostotic form predominant in 75-80% of cases [2,3]. FD can present as monostotic, affecting a single bone, or polyostotic, involving multiple bones, with the monostotic form, as seen in this case, characterized by localized skeletal involvement, contrasting with the more extensive polyostotic form, which may also associate with endocrine abnormalities in McCune-Albright syndrome [2,3]. FD commonly affects bones such as the femur, tibia, skull, and mandible, with mandibular involvement occurring in approximately 20-30% of monostotic cases [4,5]. There is no clear gender predilection, though some studies suggest a slight female predominance in craniofacial FD [2]. Clinically, FD varies from asymptomatic to symptomatic presentations, including pain, deformities, pathological fractures, and rare malignant transformation (<1% risk, increased with prior radiation) [4]. Mandibular FD often manifests as painless swelling and facial asymmetry, mimicking other fibro-osseous lesions like ossifying fibroma or neoplastic conditions such as osteosarcoma, posing significant diagnostic challenges [5].

FD’s clinical importance stems from its potential to impair function and cause complications if unrecognized. Literature documents diagnostic delays due to its subtle presentation, with cases like femoral FD misdiagnosed as osteomyelitis [6]. This report details a case of mandibular FD initially managed as chronic parotitis over seven years, reflecting the healthcare team’s best efforts to treat the patient’s symptoms with available data, and offers insights into overcoming diagnostic hurdles through multidisciplinary collaboration.

## Case presentation

An 18-year-old female patient presented with a seven-year history of recurrent right facial swelling, ultimately diagnosed as mandibular FD in October 2024. Her symptoms began in July 2017 at the age of 11, when her parents noted painless right submandibular swelling. She was evaluated in a pediatric clinic, where the team, suspecting chronic parotitis based on recurrent swelling, tenderness, and intermittent exacerbations, ordered an X-ray nasopharynx (unremarkable) and prescribed clarithromycin (15 mg/kg/day for seven days). This approach, aligned with standard parotitis management, provided temporary relief, though symptoms recurred, prompting further investigation.

**Figure 1:**
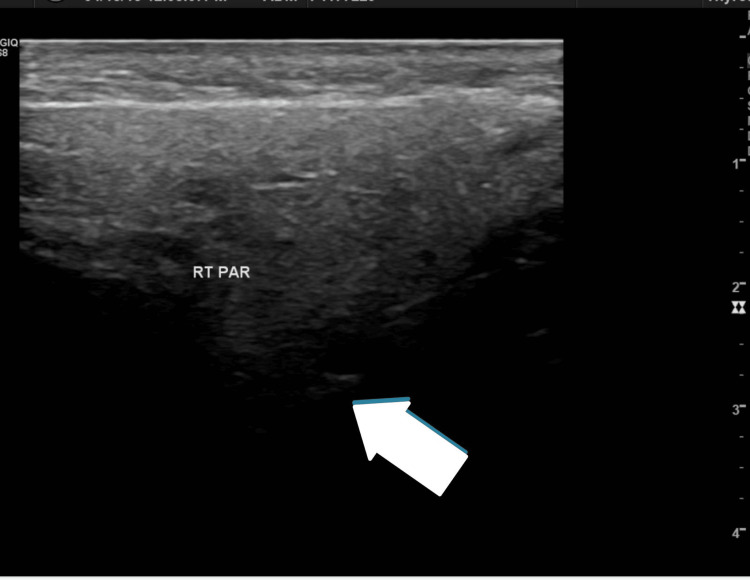
Ultrasound neck Bulky right parotid gland (4.2 cm x 2.5 cm, arrow) with homogeneous echotexture, no focal lesions, and small intra-glandular lymph nodes. RT PAR: Right Parotid

In April 2018, she presented to an ENT casualty with a two-day history of right pre-auricular swelling (approximately 3 cm x 2 cm, firm, non-tender) and trismus, without fever. The ENT team diligently reassessed her, diagnosing chronic parotitis again, and treated her with co-amoxiclav (625 mg TID for seven days) and ibuprofen (400 mg TID as needed), achieving remission. A neck ultrasound, ordered to explore salivary gland involvement, showed a bulky right parotid gland (4.2 cm x 2.5 cm) with no focal lesions and small bilateral intra-glandular lymph nodes (non-specific), supporting the working diagnosis (Figure 1).

From 2018 to 2023, the patient managed occasional mild swelling conservatively at home, with no detailed records of clinic visits, possibly due to intermittent care. In August 2023, she sought care from general surgery for persistent right submandibular swelling (4 cm x 3 cm, hard, non-tender). The team, considering sialadenitis or submandibular adenitis, ordered a second neck ultrasound, which revealed normal parotid and submandibular glands, prompting a shift in diagnostic strategy.

**Figure 2:**
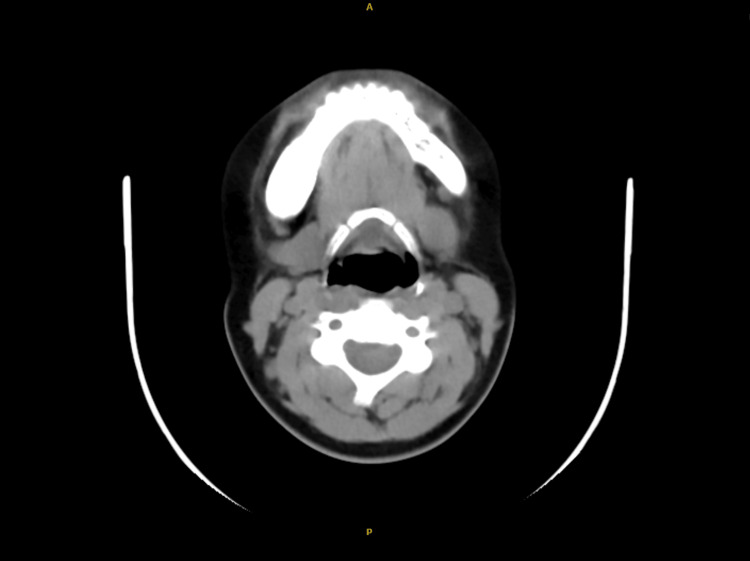
Plain neck computed tomography (CT) scan Fibrous dysplasia of the right mandible with involvement of the right alveolar foramen and oedematous changes of the right masseter muscle, sparring the right temporomandibular joint.

In October 2024, she returned to ENT with intermittent swelling (4.5 cm x 3.5 cm, firm, non-tender) and facial asymmetry. During this evaluation, an intraoral examination by the maxillofacial surgery consultant revealed no dental anomalies, malocclusion, or mucosal changes, though subtle mandibular expansion was noted on the right side, consistent with the CT findings [5]. The patient had not been previously referred to a dentist for intraoral assessment during 2017-2023, as the focus remained on salivary gland pathology [7]; however, the 2024 multidisciplinary review included this examination. The ENT team, recognizing the need for advanced imaging, ordered a CT neck without contrast, revealing an expansile mass (5 cm x 4 cm) in the right mandible with a "ground-glass" appearance, narrow transition zone, involvement of the alveolar foramen, and oedematous changes in the masseter muscle, consistent with FD (Figure 2). Histopathology was not pursued given the characteristic imaging and clinical stability.

The diagnosis of monostotic mandibular FD was confirmed by ENT in consultation with a maxillofacial surgeon based on the CT findings [8]. The patient was provided with a management plan involving multidisciplinary follow-up with maxillofacial surgery and endocrinology, including biannual CT scans to monitor progression, with potential bisphosphonates (e.g., alendronate 70 mg weekly) or surgical consultation reserved for symptom development [9,10].

## Discussion

Fibrous dysplasia (FD) is a benign fibro-osseous lesion characterized by intramedullary fibro-osseous proliferation secondary to altered osteogenesis, leading to the replacement of healthy bone with fibrous tissue and immature woven bone [1]. This case illustrates the diagnostic complexity of mandibular FD, initially managed as chronic parotitis for seven years due to overlapping clinical features and the subtlety of its presentation. FD accounts for approximately 2.5-5% of all bone lesions and 7% of benign bone tumors, with a prevalence of 1 in 10,000 to 20,000, and monostotic forms, as seen here, predominate in 75-80% of cases, with craniofacial involvement in 20-30% [2,3]. The healthcare team’s persistent efforts to address the patient’s recurrent swelling with standard treatments reflect a reasonable initial approach, yet the eventual diagnosis highlights FD’s diagnostic challenge.

Diagnosing FD can be challenging due to its clinical and radiographic similarities with other fibro-osseous lesions. In this case, the team initially diagnosed chronic parotitis based on recurrent swelling and ultrasound findings of a bulky parotid gland, criteria consistent with parotitis (pain, swelling, imaging changes) [7]. Other differentials, including ossifying fibroma (well-defined borders), cemento-osseous dysplasia (periapical location), osteosarcoma (aggressive margins), and metastatic disease (systemic signs), were not systematically excluded until the 2024 CT revealed FD’s hallmark "ground-glass" pattern, involving the alveolar foramen and masseter muscle [8]. This shift demonstrates the team’s adaptability as symptoms persisted despite temporary responses to antibiotics, a pattern also seen in a femoral FD case misdiagnosed as osteomyelitis for six months due to imaging misinterpretation [6]. The patient’s painless unilateral swelling and facial asymmetry align with mandibular FD’s typical features, underscoring the need for early skeletal consideration [5].

The seven-year diagnostic journey reflects the difficulty of identifying rare conditions with subtle presentations, not a lack of diligence. The team’s reliance on ultrasound and antibiotics was appropriate given the initial salivary gland hypothesis, with escalation to CT in 2024 showing responsiveness to unresolved symptoms [9]. Literature reports delays of months to years, reinforcing that such challenges are not unique to this case [6]. Gaps in 2018-2023 documentation suggest intermittent care rather than oversight, highlighting the importance of continuous follow-up. Radiographically, FD’s "ground-glass" appearance with poorly defined borders, as seen on CT, is a key diagnostic feature distinguishing it from other lesions, a finding the team leveraged effectively once pursued [8].

Management of FD depends on symptom severity and lesion extent. Asymptomatic cases may be monitored, while symptomatic cases may require bisphosphonates (e.g., alendronate 70 mg weekly) to alleviate pain or osteoporosis, or surgical intervention (debulking or resection) for deformity or functional impairment [10]. For this patient, the team devised a tailored plan: biannual CT scans to monitor progression, bisphosphonates if pain emerges, and surgical consultation if asymmetry worsens, balancing intervention with morbidity risks as per current guidelines [10,11]. This contrasts with earlier recommendations for extensive surgery, now tempered by higher recurrence and morbidity concerns [11].

This case advances FD understanding by demonstrating how a committed team navigated a rare diagnosis initially masked as a common condition, advocating for advanced imaging (e.g., CT) in persistent swelling cases [8]. Compared to the literature, it aligns with diagnostic challenges but uniquely emphasizes mandibular FD’s subtlety and the value of persistent clinical reassessment [9]. It underscores the need for clinician awareness of FD in craniofacial differentials, enhancing diagnostic accuracy through multidisciplinary collaboration.

## Conclusions

This case of mandibular FD, initially managed as chronic parotitis over seven years, showcases the healthcare team’s diligent efforts to address a complex presentation, culminating in an accurate diagnosis through advanced imaging. Key lessons include the importance of considering FD in persistent facial swellings and the effectiveness of escalating diagnostic tools when standard approaches falter. We recommend clinician education on FD’s craniofacial signs, its routine inclusion in differential diagnoses, and multidisciplinary teamwork to refine diagnostic accuracy. Future efforts should focus on standardized protocols to support teams in recognizing FD early, optimizing patient outcomes.
